# Inhibition of Tropomyosin Receptor Kinase B Signaling Alters Hepatic Mitochondrial Enzyme Activity and Oxidative Stress in Mice With Sucrose‐Induced Insulin Resistance

**DOI:** 10.1002/jbt.71104

**Published:** 2026-08-31

**Authors:** Akın Bodur, Hüseyin Çınar Zihni, Katip Korkmaz, Solmaz Bodur, Ahmet Alver

**Affiliations:** ^1^ Department of Nutrition and Dietetics, Faculty of Health Sciences Kahramanmaraş İstiklal University Kahramanmaraş Türkiye; ^2^ Department of Nutrition and Dietetics, Faculty of Health Sciences Kahramanmaraş Sütçü İmam University Kahramanmaraş Türkiye; ^3^ Department of Medical Biochemistry, Graduate School of Health Sciences Karadeniz Technical University Trabzon Türkiye; ^4^ Department of Nutrition and Dietetics, Faculty of Health Sciences Karadeniz Technical University Trabzon Türkiye; ^5^ Department of Medical Biochemistry, Faculty of Medicine Adıyaman University Adıyaman Türkiye; ^6^ Department of Medical Biochemistry, Faculty of Medicine Karadeniz Technical University Trabzon Türkiye

**Keywords:** BDNF, insulin resistance, mitochondrial enzyme activity, oxidative stress, TrkB

## Abstract

BDNF and its receptor, TrkB, are essential regulators of energy homeostasis in both central and peripheral tissues, including the liver. This study aimed to investigate the effects of ANA‐12, a selective TrkB antagonist, on hepatic mitochondrial enzyme activities and biochemical parameters in a mouse model of sucrose‐induced insulin resistance. Male C57BL/6J mice were divided into four groups: Control, Sucrose (35% solution), Sucrose+DMSO, and Sucrose+ANA‐12 (0.5 mg/kg). Following a 16‐week dietary intervention, ANA‐12 was administered via intraperitoneal injection during the final 21 days. Hepatic activities of cytochrome c oxidase, superoxide dismutase (SOD), and catalase (CAT), along with malondialdehyde (MDA) levels, were analyzed to evaluate mitochondrial enzyme activity and oxidative stress‐related biochemical changes. Chronic sucrose consumption significantly increased body weight, serum insulin levels, HOMA‐IR scores, and cholesterol levels. While ANA‐12 treatment showed a numerical tendency to attenuate weight gain and HOMA‐IR scores, these changes did not reach statistical significance. However, the ANA‐12 group exhibited significantly lower serum leptin and ceramide levels compared to the Sucrose+DMSO group. In the liver, sucrose consumption triggered oxidative stress, as evidenced by increased MDA levels. Conversely, TrkB inhibition was associated with increased cytochrome c oxidase activity, altered SOD activity, and reduced hepatic MDA concentrations compared with the Sucrose+DMSO group, suggesting partial modulation of oxidative stress‐related biochemical alterations. Furthermore, chronic sucrose consumption was associated with reduced hepatic BDNF levels. Overall, these findings indicate that TrkB inhibition may influence selected hepatic mitochondrial enzyme activities and redox‐related markers under sucrose‐induced metabolic stress. However, these results should be interpreted cautiously because systemic insulin resistance markers were not significantly improved, and additional control groups are required to clarify ANA‐12‐specific and vehicle‐related effects.

## Graphical abstract

Suppression of BDNF‐TrkB signaling has shown a protective trend on hepatic oxidative stress by reducing sucrose‐induced lipid peroxidation. BDNF–TrkB signaling pathway plays a peripheral role in regulating mitochondrial enzyme capacity and redox balance in the liver.

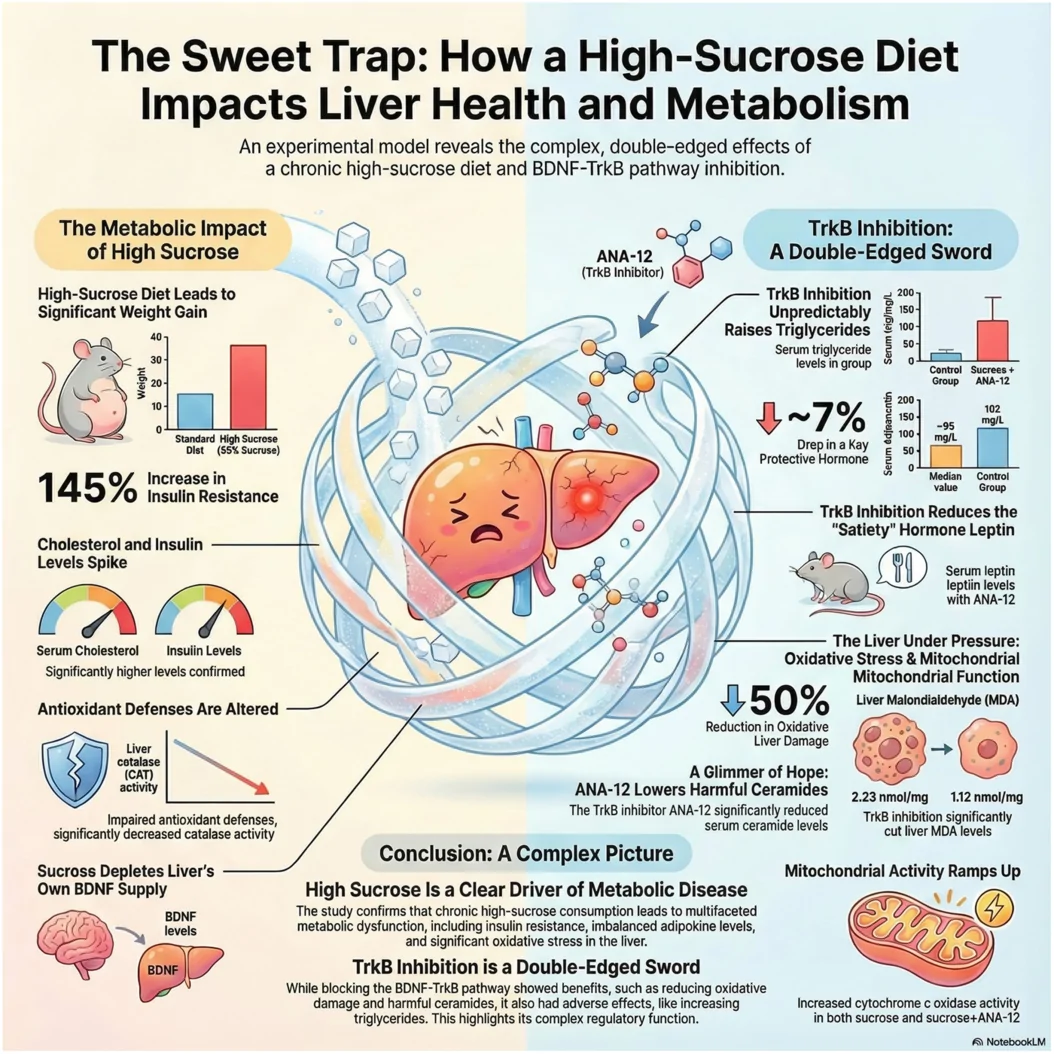

## 1 Introduction

Brain‐derived neurotrophic factor (BDNF) is a key neurotrophic molecule that plays a fundamental role in the development, maintenance, and plasticity of both the central and peripheral nervous systems. It supports the differentiation of neurons derived from stem cells, promotes synaptogenesis, and exerts anti‐apoptotic effects. Although BDNF is predominantly synthesized in neuronal cells, both BDNF and its high‐affinity receptor, tropomyosin receptor kinase B (TrkB), are also expressed in several peripheral tissues, including skeletal muscle, cardiac tissue, liver, and adipose tissue [1, 2]. TrkB serves as a critical mediator of activity‐dependent neuronal plasticity and functions as the primary receptor for BDNF [3]. The coordinated interaction between BDNF and TrkB contributes to the regulation of energy homeostasis and glucose metabolism across distinct brain regions, thereby influencing energy expenditure through both homeostatic and hedonic feeding mechanisms [4]. In contrast, the p75 neurotrophin receptor (p75NTR), another major receptor involved in BDNF signaling, has been shown to function antagonistically to TrkB in the regulation of feeding behavior. While TrkB signaling predominantly suppresses food intake, p75NTR promotes feeding, acting as a counter‐regulatory mechanism under conditions of severe energy deficiency [5]. Accordingly, deficiencies in BDNF expression or disruptions in BDNF signaling pathways have been implicated in the development of hyperphagia and obesity in both humans and experimental animal models [6]. Among the two major forms of BDNF, mature BDNF preferentially binds to TrkB and stimulates the expression of proteins involved in mitochondrial biogenesis. BDNF enhances the synthesis of antioxidant proteins while suppressing proapoptotic signaling. Furthermore, BDNF promotes mitochondrial biogenesis through activation of the PKA/CREB/PGC‐1α signaling pathway following TrkB engagement. Although antioxidant systems do not directly induce mitochondrial biogenesis, they contribute indirectly by strengthening cellular defense mechanisms against oxidative stress in response to BDNF signaling [7]. ANA‐12 is a selective TrkB antagonist that inhibits BDNF‐induced TrkB activation. The inhibitory effect of ANA‐12 on the BDNF–TrkB signaling pathway was first demonstrated by Cazorla et al. [8]. Since then, this compound has been increasingly employed to investigate the role of BDNF–TrkB signaling across various tissues and molecular pathways. Importantly, the absence of significant adverse effects associated with ANA‐12 has further facilitated its experimental and translational applications [9].

Insulin resistance is a pathological condition in which the responsiveness of cells and tissues to insulin is reduced. This condition represents a major risk factor for the development of several metabolic disorders, including type 2 diabetes mellitus (T2DM), cardiovascular diseases, and non‐alcoholic fatty liver disease. Therefore, elucidating the molecular and cellular mechanisms underlying insulin resistance may play a critical role in identifying effective strategies to prevent or mitigate T2DM and related metabolic complications. Emerging evidence suggests that neurotrophic signaling pathways, including BDNF–TrkB, may intersect with these metabolic processes [10, 11].

The modulation of insulin resistance is influenced by both genetic and environmental factors. Genetic determinants include mutations and polymorphisms in insulin receptors as well as in key signaling molecules such as insulin receptor substrates (IRS) and phosphoinositide 3‐kinase. The coordinated interaction of these components, particularly through the activation of IRS, represents a central mechanism linking insulin receptor activation to downstream metabolic responses. Environmental contributors include elevated circulating metabolites, notably chronically increased free fatty acid levels associated with lipotoxicity, as well as inflammatory mediators, the gut microbiota, and the extent of obesity. In insulin‐resistant states, serine phosphorylation of IRS‐1 is markedly increased, and hyperphosphorylation at these serine residues is widely recognized as a negative regulator of insulin signaling [12].

A dietary sucrose–induced model of insulin resistance has been established in rodent species, including rats and mice, using sucrose administered in either solid or liquid form. These experimental models have been widely employed to elucidate the pathophysiological mechanisms underlying insulin resistance and its associated metabolic disturbances, such as oxidative stress and inflammation [13, 14]. Notably, recent evidence indicates that liquid sucrose consumption leads to more pronounced increases in body weight, greater impairment of glucose tolerance, and enhanced hepatic lipid accumulation compared with an isocaloric solid sucrose diet [15]. Among the biochemical mechanisms contributing to insulin resistance, mitochondrial dysfunction plays a pivotal role. Mitochondria are the primary cellular sites of reactive oxygen species (ROS) generation, and excessive mitochondrial ROS accumulation has been shown to impair glucose tolerance and exacerbate insulin resistance [16].

Although the central roles of BDNF and TrkB in energy homeostasis are well established, their tissue‐specific effects on hepatic mitochondrial function under insulin‐resistant conditions remain poorly understood. In particular, the consequences of pharmacological TrkB inhibition on liver mitochondrial enzyme activity and oxidative stress in sucrose‐induced insulin resistance have not been sufficiently investigated. Therefore, elucidating the effects of TrkB receptor inhibition on hepatic mitochondrial function and redox homeostasis is essential for advancing our understanding of the peripheral metabolic actions of BDNF signaling.

## 2 Material and Methods

All experimental procedures were approved by the Karadeniz Technical University Animal Experiments Local Ethics Committee (Approval No. 2024/20) and were performed at the Karadeniz Technical University Surgical Application and Research Center. The study was conducted using male C57BL/6 J mice aged 4–6 weeks (body weight: 10–15 g), obtained from the Karadeniz Technical University Surgical Practice and Research Center. Animals were housed in standard laboratory cages under controlled environmental conditions, with ad libitum access to food and water, and maintained on a 12‐h light/dark cycle throughout the experimental period. All experimental protocols were conducted in accordance with the National Institutes of Health guidelines for the care and use of laboratory animals and were designed in compliance with the ARRIVE guidelines to ensure transparency, rigor, and reproducibility in animal research [17, 18]. Animal health and well‐being were routinely monitored throughout the study to ensure welfare standards and data reliability.

### 2.1 Experimental Design and Animal Grouping

The sample size was calculated using the G*Power program. Based on an effect size of 0.80, an alpha error of 0.05, a power (1–β) of 0.95, and four groups, the total sample size was determined to be 32. Accordingly, eight subjects were randomly assigned to each group. The subjects in each group were placed in separate cages and positioned so that they could not touch each other. All groups were maintained for a total duration of 16 weeks under standard laboratory conditions. *Group I (Control)*: Animals in this group were fed a standard laboratory‐type rodent chow (LTRC) that did not contain sucrose for 16 weeks. *Group II (Sucrose Solution)*; animals in this group were fed only with LTRC during the first 2 weeks. Subsequently, they were provided with free access to a 35% sucrose solution (35 g sucrose/100 mL tap water) in addition to LTRC, without access to any other drinking water, for an additional 14 weeks, completing a total feeding duration of 16 weeks. *Group III (Sucrose Solution* + *DMSO)*; animals in this group were maintained on LTRC alone for the first 2 weeks. Thereafter, they were given free access to a 35% sucrose solution (35 g sucrose/100 mL tap water) together with LTRC for 14 weeks, with no access to alternative drinking water. During the final 21 days of the feeding period, all mice received daily intraperitoneal (i.p.) injections of 17% dimethyl sulfoxide (DMSO). *Group IV (Sucrose Solution* + *ANA‐12)*; animals in this group were fed with LTRC alone for the first 2 weeks, followed by a 14‐week period of free access to a 35% sucrose solution (35 g sucrose/100 mL tap water) in addition to LTRC, without any other water source. During the final 21 days of the feeding period, all mice received an i.p. injection of ANA‐12 at a dose of 0.5 mg/kg, dissolved in 17% DMSO, according to the method described by Zhang et al. (2015) [19]. This design was intended to evaluate the effects of subchronic ANA‐12 administration after the establishment of chronic sucrose‐induced metabolic stress, rather than to model continuous TrkB inhibition throughout the full dietary intervention period. Throughout the feeding period, the body weights of the mice were measured and recorded every 14 days starting from the beginning of the experiment.

### 2.2 Drug Preparation and Administration

ANA‐12 (product number A327589, 99.47% purity, Ambeed, USA) was freshly prepared before each administration. The compound was initially solubilized in 17% DMSO (Lot No. 49H3641, Sigma‐Aldrich, USA) and subsequently diluted with phosphate‐buffered saline (PBS, pH 7.4) to obtain the desired working concentration, in accordance with the protocol reported by Liu et al. [20]. The solution was administered i.p. at a dose of 0.5 mg/kg once daily for 21 consecutive days. The same volume of vehicle (17% DMSO in PBS) was injected into the corresponding control group. All injections were performed at the same time each day to minimize circadian variability.

Upon the conclusion of the experimental duration, the subjects were subjected to anesthesia via ketamine (80 mg/kg) and xylazine (10 mg/kg), followed by euthanasia through decapitation. Rodents were sacrificed through decapitation after a period of food deprivation, and trunk blood was subsequently collected into tubes containing separator gel, from which blood sera were subsequently extracted. Serum and liver tissues were preserved at –80°C prior to conducting experimental evaluations.

### 2.3 Measurement of Serum Biochemical Parameters

Serum samples obtained from experimental subjects were analyzed for amylase, as well as for aspartate aminotransferase (AST) and alanine aminotransferase (ALT) to evaluate hepatic and pancreatic functions, and for glucose, triglyceride, and total cholesterol to assess overall metabolic status. These analyses were performed using a Beckman Colter autoanalyzer at the KTU Farabi Application and Research Center Clinical Biochemistry Laboratory through a service procurement arrangement. Serum glucose, TG, cholesterol, and amylase concentrations were expressed as mg/dL. Serum AST and ALT activities expressed as U/L.

### 2.4 Measurement of Serum and Liver BDNF Levels

The quantification of serum and liver BDNF (Catalog number: E0013Mo, BT LAB, China) concentrations in serum was conducted utilizing commercially available enzyme‐linked immunosorbent assay (ELISA) kits. Liver tissue homogenate and serum measurements were conducted in alignment with the specifications provided by the manufacturer. Total protein concentrations were quantified utilizing a commercially available bicinchoninic acid assay kit (BCA) (Catalog Number: E‐BC‐K318‐M; Elabscience, USA) in accordance with the guidelines provided in the manufacturer's instruction manual. This method relies on the biuret reaction, in which Cu^2+^ ions are reduced to Cu^+^ by proteins under alkaline conditions, followed by the formation of a purple‐colored Cu^+^–BCA complex that can be quantified at 562 nm. Total protein concentrations in liver homogenates were quantified and reported in mg/mL. BDNF concentrations in serum were reported in ng/mL, whereas liver BDNF levels were normalized to total protein content and presented as ng/mg protein.

### 2.5 Measurement of Serum Insulin, Adiponectin, Leptin, and Ceramide Levels

The measurement of insulin (Catalog number: E‐EL‐M2614; Elabscience, USA), adiponectin (Catalog number: E0246Mo, BT LAB, China), leptin (Catalog number: E0652Mo, BT LAB, China), and ceramide (Catalog number: EA0130Mo, BT LAB, China) levels in serum was performed using standardized ELISA kits that are readily available commercially. The Homeostatic Model Assessment for Insulin Resistance (HOMA‐IR) was calculated based on fasting insulin and glucose concentrations using the (HOMA‐IR = [Insulin (µU/mL) × Fasting Glucose (mmol/L)]/22.5) formula [21]. Besides, the leptin/adiponectin ratio was calculated. Serum insulin, adiponectin, leptin, and ceramide levels were expressed as ng/mL, mg/L, ng/L, and µmol/L, respectively.

### 2.6 Measurement of Liver Cytochrome c Oxidase, Superoxide Dismutase (SOD), and Catalase (CAT) Activities

Cytochrome c oxidase is one of the key enzymes in the mitochondrial respiratory chain. It catalyzes the oxidation of reduced cytochrome c, generated by mitochondrial complex III, and consumes molecular oxygen to produce water. Cytochrome c oxidase activity was determined by measuring absorbance changes at 550 nm associated with the oxidation of the reduced form of the enzyme. Cytochrome c oxidase activity in liver tissues was determined using a commercially available assay kit (Catalog number: E‐BC‐K152‐M, Elabscience, USA) according to the manufacturer's instructions. Approximately 100 mg of liver tissue was homogenized in 900 µL of extraction solution A at +4°C. Following homogenization, samples were first centrifuged at 600 × g for 5 min. The supernatant obtained was subsequently subjected to a second centrifugation at 11,000 × g for 10 min at 4°C. The supernatant obtained from this step was discarded. The resulting pellet was resuspended in 200 µL of extraction solution B containing 10 µL of inhibitor, followed by sonication for 1 min. Afterward, the sample was centrifuged at 11,000 × g, and the final supernatant obtained was used for enzyme activity measurements.

SOD and CAT activities were assessed in liver tissue samples. SOD activity was determined using a modified protocol originally described by Sun and Oberley (1988) [22], which is based on the inhibition of nitroblue tetrazolium (NBT) reduction by superoxide anions (O_2_•^−^) generated in the xanthine–xanthine oxidase system. The formation of purple formazan was quantified spectrophotometrically at 560 nm. Catalase activity was measured according to the method described by Aebi (1984) [23], which monitors the decrease in absorbance at 240 nm resulting from the enzymatic decomposition of hydrogen peroxide (H_2_O_2_). Protein concentrations in mitochondrial fractions were quantified using the bicinchoninic acid (BCA) assay (Catalog No. E‐BC‐K318‐M; Elabscience, USA). Cytochrome c oxidase and SOD activities were expressed as units per milligram of protein (U/mg protein), whereas CAT activity was expressed as k/g protein.

### 2.7 Measurement of Liver Malondialdehyde (MDA) Levels

Hepatic oxidative stress was evaluated by determining MDA concentrations. Approximately 50 mg of liver tissue was used for MDA analysis. Tissue samples were homogenized at 4°C in a buffer containing 1.15% KCl supplemented with Triton X‐100 (0.5 mL/L). The homogenates were subsequently centrifuged at 3000 rpm for 10 min, and the resulting supernatants were collected for analysis. MDA levels were quantified based on their reaction with thiobarbituric acid under acidic conditions, forming a pink chromogenic complex that was measured spectrophotometrically at 532 nm. MDA concentrations were normalized to total protein content and expressed as nmol MDA per mg protein, in accordance with the method described by Mihara et al. [24].

### 2.8 Statistical Analyses

Body weight results are summarized as the mean with the corresponding standard error of the mean (SEM). All other variables are presented as median with interquartile range (IQR; 25th–75th percentiles). Graphical representations were generated using box‐and‐whisker plots. Between‐group differences were assessed using the non‐parametric Kruskal–Wallis test. Following the detection of a statistically significant overall effect, post hoc pairwise comparisons were undertaken using the Mann–Whitney *U* test. A threshold of *p* < 0.05 was applied to determine statistical significance.

## 3 Results

### 3.1 Temporal Pattern of Weight Gain

During the 16‐week dietary intervention period, a significant increase in body weight was observed in the sucrose and sucrose + DMSO groups compared to the control group (*p* < 0.05). The control animals maintained relatively stable body weights throughout the study, whereas sucrose‐fed animals exhibited a progressive increase, particularly evident after the 8 week. The co‐administration of DMSO with sucrose did not significantly alter the magnitude of weight gain compared to the sucrose‐only group, indicating that the observed changes were primarily attributable to sucrose intake rather than solvent effects. Co‐administration of ANA‐12 with sucrose resulted in body weight values that were numerically lower than those observed in the sucrose group and closer to those of the control group; however, this difference did not reach statistical significance. Thus, while ANA‐12 treatment showed a tendency toward attenuation of sucrose‐induced weight gain, no statistically significant difference was detected between the ANA‐12 group and either the control or sucrose groups.

These results suggest that chronic exposure to a high‐sucrose solution leads to measurable body weight elevation in experimental animals, supporting the role of excessive dietary sugar in promoting weight gain under sustained consumption conditions.

### 3.2 Effects of Sucrose, DMSO, and ANA‐12 on Glycemic Control, Lipid Metabolism, and Insulin Resistance

No statistically significant difference was detected in fasting serum glucose levels among the groups (*p* > 0.05). However, it was observed that the median values in the sucrose+DMSO and sucrose + ANA‐12 groups were higher compared to the control and sucrose groups. A significant elevation in serum cholesterol was observed in every sucrose‐consuming group when compared to the control group (*p* < 0.05). The serum TG levels of the sucrose and sucrose+DMSO groups were found to be similar to the control group, and no statistically significant difference was observed (*p* > 0.05). Compared to both the control and sucrose groups, mice receiving sucrose in combination with ANA‐12 showed a significant increase in serum triglyceride levels (*p* < 0.05). Conversely, sucrose consumption led to a significant reduction in serum ALT levels in all sucrose‐treated groups when compared with controls (*p* < 0.05). In line with the ALT results, serum AST concentrations were significantly reduced in both the sucrose and sucrose+DMSO groups when compared with controls (*p* < 0.05). In all sucrose‐consuming groups, serum insulin levels were found to be significantly higher compared to the control group (*p* < 0.05). The highest insulin level was observed in the sucrose + DMSO group. The serum insulin levels of the sucrose + ANA‐12 group were numerically lower compared to the sucrose+DMSO group. However, the observed difference was not statistically significant (*p* > 0.05). HOMA‐IR scores in all sucrose‐consuming groups were found to be statistically significantly higher compared to the control group (*p* < 0.05). Serum amylase levels of the sucrose group were numerically lower compared to the control group. However, this difference did not reach statistical significance (*p* > 0.05). In contrast, the sucrose+DMSO and sucrose+ANA‐12 groups exhibited significantly reduced amylase levels relative to the control group (*p* < 0.05).

### 3.3 Alterations in Adipokines, Ceramide, and BDNF Levels Following DMSO and ANA‐12 Treatment

Serum leptin concentrations were significantly elevated in the sucrose+DMSO group relative to the sucrose‐only group (*p* < 0.05). Notably, the sucrose + ANA‐12 group demonstrated significantly lower serum leptin levels compared to both the control group and the sucrose + DMSO group (*p* < 0.05). All sucrose‐treated groups displayed significantly reduced serum adiponectin levels compared to the control group (*p* < 0.05). The Sucrose + DMSO group exhibited a significantly elevated leptin/adiponectin ratio compared to both the control and sucrose groups (*p* < 0.05). The Sucrose + ANA‐12 group showed a higher leptin/adiponectin ratio, which was significantly higher than the control group (*p* < 0.05), but lower than the sucrose+DMSO group. The Sucrose + DMSO group showed dramatically elevated serum ceramide levels compared to both the control and sucrose groups (*p* < 0.05). Remarkably, the sucrose+ANA‐12 group exhibited significantly lower serum ceramide levels compared to the sucrose+DMSO group (*p* < 0.05). Serum BDNF levels showed significant reductions in both DMSO and ANA‐12‐treated groups. The Sucrose+DMSO group demonstrated significantly lower serum BDNF levels compared to both the control and sucrose groups (*p* < 0.05). Similarly, the sucrose+ANA‐12 group showed reduced serum BDNF levels compared to the sucrose group (*p* < 0.05). In the sucrose‐treated group, hepatic BDNF levels were measured at 1.70 ng/mL, demonstrating a statistically significant reduction compared to the control group (*p* < 0.05). In the sucrose+ANA‐12 group, BDNF levels were determined to be, also showing a statistically significant decrease compared to the control group (*p* < 0.05).

### 3.4 Effects of Sucrose, DMSO, and ANA‐12 on Mitochondrial Enzyme Activity and Oxidative Stress Markers

Cytochrome c oxidase activity significantly increased in the sucrose group compared to the control group (*p* < 0.05). In the sucrose+DMSO group, cytochrome c oxidase activity was measured at 89.58 U/mg protein, while the sucrose+ANA‐12 group showed the highest value (198.33 U/mg protein, *p* < 0.05 vs. control and sucrose+DMSO). SOD activity was 42.07 U/mg protein in the control group and significantly increased in all treatment groups (*p* < 0.05). Catalase activity was 270.02 k/g protein in the control group but significantly decreased in all treatment groups (*p* < 0.05). MDA levels increased significantly in the sucrose group compared to the control (*p* < 0.05). In contrast, the sucrose+DMSO group and especially the sucrose+ANA‐12 group showed a significant reduction compared with the sucrose group (*p* < 0.05).

## 4 Discussion

In this study, experimental insulin resistance was first induced using a 35% sucrose solution for 16 weeks, and TrkB receptor inhibition was achieved through ANA‐12 administration. In this context, hepatic mitochondrial enzyme activity and oxidative stress‐related biochemical changes were evaluated together with selected metabolic and adipokine‐related parameters.

**Figure 1:**
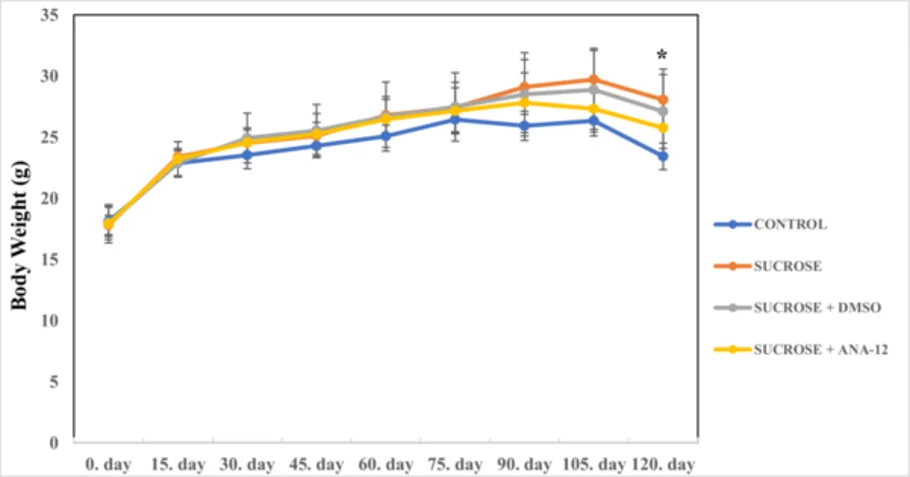
Weight alterations observed throughout the duration of 4‐month dietary interventions. *p < 0.05 in relation to the sucrose and sucrose+DMSO groups with control.

As a result of the 16‐week feeding period, body weight increased significantly in the sucrose group compared to the control. In addition, the body weight of the sucrose + DMSO group has also increased significantly compared to the control group. Accordingly, the sucrose solution contributed approximately twice as much to the increase in body weight. Furthermore, the solvent did not contribute significantly to body weight. However, ANA‐12 reduced the increase in body weight induced by sucrose, but this reduction was not statistically significant (Table 1, Figure 1). A 35% sucrose solution administered over a 28‐day period was demonstrated to increase body weight in male and female Wistar rats, whereas no significant effect of DMSO on body weight was observed in the same study [25]. It has been demonstrated that consumption of a 30% liquid sucrose diet for 12 weeks increases body weight in male C57BL/6 J mice [14]. A 9‐week intake of a 35% sucrose solution was not associated with any significant body‐weight variation in Wistar rats [26]. In a comparable manner, body weight did not differ significantly between rats receiving a 35% sucrose solution for 9 weeks and those in the control group [27]. Furthermore, a recent study has shown that the ANA‐12 application does not significantly alter body weight. However, previous studies have shown that the ANA‐12 application plays an important role in controlling body lipid accumulation when mice are fed a cafeteria diet [9].

**Table 1:** Initial and final body weights and fasting serum glucose, cholesterol, TG, ALT, AST, insulin levels, and HOMA‐IR score of control, sucrose, sucrose+DMSO, and sucrose+ANA‐12 groups for 16 weeks.

	Control	Sucrose	Sucrose + DMSO	Sucrose + ANA‐12
Initial body weight, *g* *	18.10 ± 0.4	17.81 ± 0.28	17.95 ± 0.46	17.91 ± 0.55
Final body weight, *g* *	23.43 ± 0.39	28.05 ± 0.89^a^	27.10 ± 1.14^a^	25.76 ± 0.87
Glucose, mg/dL**	148 (20) [141–161]	144 (20) [136–156]	166 (53) [132–185]	164 (48) [145–193]
Cholesterol, mg/dL**	68 (12) [64–76]	114 (25)^a^ [105–130]	106 (35)^a^ [85–120]	113 (34)^a^ [93–127]
TG, mg/dL**	94 (17) [82–99]	96 (14) [86–100]	90 (26) [84–110]	106 (21)a,b [100–121]
ALT, U/L**	52 (16) [48–64]	20 (3)^a^ [20–23]	24 (7)^a^ [20–27]	30 (12)^a^, ^b^ [24–36]
AST, U/L**	264 (168) [228–396]	160 (48)^a^ [133–181]	156 (48)^a^ [128–176]	196 (52)^a^, ^b^, ^c^ [184–236]
Insulin, ng/mL**	0.75 (1.02) [0.49–1.50]	1.74 (1.09)^a^ [1.11–2.20]	2.13 (0.96)^a^ [1.76–2.71]	2.04 (1.32)^a^ [1.34–2.66]
HOMA‐IR Score**	7.67 (10.04) [5.03–15.07]	18.79 (10.93)^a^ [13.40–24.34]	28.33 (24.92)^a^ [15.30–40.22]	20.95 (19.87)^a^ [13.39–33.26)
Amylase, mg/dL**	3638 (486) [3340–3826]	3094 (970) [2796–3766]	3092 (924)^a^ [2426–3350]	3084 (1706)^a^ [2857–4563]

^a^ *p* < 0.05 compared with control.

^b^ *p* < 0.05 compared with sucrose.

^c^ *p* < 0.05 compared with sucrose + DMSO.

* Values are means ± SE.

** Values are median – (IQR) and quartiles of 25%–75%.

It has been determined that chronic sucrose solution feeding may lead to suppression of pancreatic exocrine function and that ANA‐12 has no clear effect on serum amylase levels. However, treatment with DMSO causes a slight decrease in pancreatic function (Table 1). In our study, feeding with a chronic consumption of sucrose solution led to hypercholesterolemia consistent with a previous study [28]. However, ANA‐12 administration was insufficient against hypercholesterolemia. In our study, high sucrose solution consumption during the chronic period had no significant effect on fasting serum TG levels. Notably, TrkB inhibition was found to potentially cause impairment in TG metabolism (Table 1).

Interestingly, in our study, consumption of a 35% sucrose solution led to a decrease in serum ALT and AST levels, and TrkB antagonism was found to partially block this observed effect of sucrose (Table 1). In preclinical studies involving high sucrose diets, serum ALT and AST levels are generally observed to increase [28, 29]. However, da Silva and colleagues found that a high sucrose diet reduced serum ALT levels in subjects with liver cirrhosis caused by thioacetamide, demonstrating a protective effect on the liver and mitigating catabolic effects [30]. In another study, it was found that pyridoxine deficiency caused by insufficient solid food intake due to 30% sucrose consumption led to a decrease in ALT and AST activity [31].

Serum glucose levels remained statistically similar between groups; nevertheless, sucrose intake led to a significant increase in serum insulin levels across all treated groups. This study found that hyperinsulinemia conditions led to higher HOMA‐IR scores, which were designated as significant development of insulin resistance in all groups containing sucrose. Interestingly, the highest HOMA‐IR score was detected in the group receiving DMSO. Although the ANA‐12 application caused a decrease in the HOMA‐IR score, this decrease was not statistically significant (Table 1). It has been demonstrated that DMSO enhances insulin‐induced GLUT4 translocation to the adipocyte cell surface [32]. A recent review states that dysfunction of intracellular GLUT4‐containing vesicles is among the primary causes of systemic insulin resistance [33]. In addition, DMSO is known to induce the movement of GLUT4 towards the cell membrane [34]. In another study, although DMSO reduced glucose and body weight, it was noted that the therapeutic effect of 7,8‐dihydroxyflavone, a BDNF mimetic, was stronger than that of DMSO [35]. It has also been suggested that DMSO may exert its effect by increasing gluconeogenesis and reducing mitochondrial functions [36]. The distinct biochemical profile observed in the Sucrose+DMSO group should be considered when interpreting the ANA‐12‐related findings. In the present study, DMSO administration in sucrose‐fed mice was associated with marked changes in several parameters, including increased ceramide levels, altered HOMA‐IR scores, and reduced amylase levels. These findings suggest that DMSO may not have acted as a completely inert vehicle under the present sucrose‐induced metabolic conditions. Although comparison of the Sucrose+ANA‐12 group with the Sucrose+DMSO group partially controls for vehicle exposure, the absence of a DMSO‐alone group under normometabolic conditions prevents a clear assessment of the basal metabolic effects of DMSO. Therefore, the observed ANA‐12‐related changes should be interpreted cautiously, considering that vehicle‐related effects may have contributed to the overall biochemical profile.

Consistent with our findings, Burgeiro et al. reported that rats consuming a 35% sucrose solution for 9 weeks did not exhibit a significant change in fasting serum glucose levels compared with controls; however, serum insulin concentrations and HOMA‐IR indices were significantly increased. They pointed out that even though fasting glucose concentrations are maintained within the standard normative range (normoglycemia), the existence of compensatory hyperinsulinemia signifies a developing level of glucose intolerance and peripheral insulin resistance [27]. Additionally, it has been determined that liquid sucrose consumption causes disturbances in glucose homeostasis [15, 28]. A prediabetic state characterized by impaired glucose tolerance and high serum insulin levels was induced in rats fed a 30% sucrose solution for 14 days [37]. Obesity and impaired glucose tolerance were observed in male C57BL/6J mice fed a 30% sucrose solution for 12 weeks [14]. Furthermore, in dietary studies conducted in rodents using DMSO as a solvent, it has been observed that DMSO does not contribute to the development of insulin resistance, or that this aspect of the research has not been emphasized in detail [38, 39, 40].

**Figure 2:**
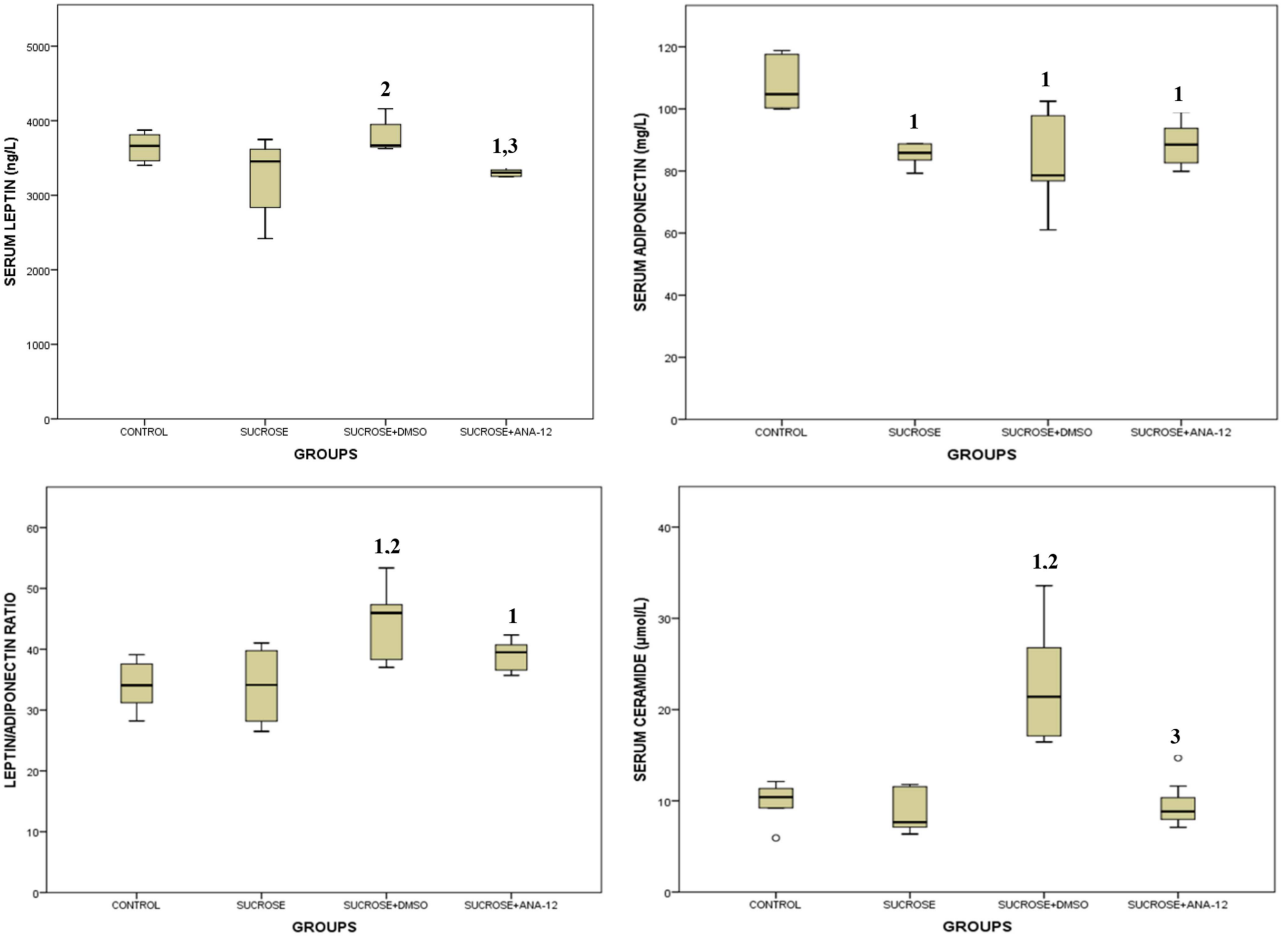
Serum leptin, adiponectin, and ceramide levels and leptin/adiponectin ratio in each group. (^1^ *p* < 0.05 compared with control; ^2^ *p* < 0.05 compared with sucrose; ^3^ *p* < 0.05 compared with sucrose+DMSO).

Leptin is a hormone that plays an important role in appetite control and contributes to the development of eating disorders [41]. Our study has determined that TrkB inhibition has a reducing effect on serum leptin levels. However, feeding with a high sucrose solution had no effect on serum leptin levels (Table 2, Figure 2). A study conducted on rats who fed a high‐sucrose diet showed that liquid sucrose consumption can lead to leptin resistance [42]. Besides, a recent meta‐analysis examining leptin and diabetes together emphasized that high serum leptin levels are not associated with T2DM [43]. Another study showed that a sucrose‐rich diet causes hyperleptinemia in Wistar rats [44]. Rats consuming a 30% sucrose solution exhibited significantly elevated serum leptin concentrations after 6 months compared with controls [45].

**Table 2:** Serum leptin, adiponectin, ceramide, and BDNF levels and leptin/adiponectin ratio of control, sucrose, sucrose+DMSO, and sucrose+ANA‐12 groups for 4 months.

	Control	Sucrose	Sucrose + DMSO	Sucrose + ANA‐12
*Leptin (ng/L)*	3662.12 (427.55) [3418.22–3845.77]	3453.37 (842.90) [2786.35–3629.24]	3669.54 (386.78)^b^ [3638.45–4025.23]	3303.44 (96.13)^a^, ^c^ [3250.48–3346.60]
*Adiponectin (mg/L)*	102.04 (17.22) [97.37–114.59]	95.13 (28.91)^a^ [84.13–113.04]	97.82 (30.94)^a^ [76.82–107.76]	92.50 (14.66)^a^ [84.92–99.58]
*Leptin/Adiponectin Ratio*	35.36 (9.04) [29.92–38.96]	34.13 (13.79) [26.92–40.71]	42.32 (13.11)^a^, ^b^ [36.60–49.71]	37.30 (7.79)^a^ [33.38–41.17]
*Ceramide (µmol/L)*	10.92 (4.84) [9.49–14.33]	7.36 (5.33) [5.27–10.60]	16.78 (14.21)^a^, ^b^ [8.91–23.12]	7.11 (3.66)^c^ [5.42–9.08]
*BDNF (ng/mL)*	11.42 (0.86) [10.75–11.61]	11.72 (1.37) [10.87–12.23]	10.21 (1.10)^a^, ^b^ [9.21–10.31]	10.34 (1.03)^b^ [10.21–11.25]

*Note:* All values are expressed as median – (IQR) and quartiles of 25%–75%.

^a^ *p* < 0.05 compared with control.

^b^ *p* < 0.05 compared with sucrose.

^c^ *p* < 0.05 compared with sucrose+DMSO.

Adiponectin is an adipokine that plays an important role in metabolic regulation, particularly in relation to obesity and diabetes. Plasma adiponectin levels are influenced by factors such as diet type, BDNF levels, and the localization of adipose tissue [46, 47]. Additionally, this hormone contributes to glucose homeostasis [48]. In our study, serum adiponectin levels decreased in all groups containing sucrose compared to the control group. However, ANA‐12 administration had no effect on serum adiponectin levels (Table 2, Figure 2). Serum adiponectin levels in C57BL/6 mice fed a 32% sucrose diet for 8 weeks were shown to be significantly reduced compared to controls [49]. Adipor1 gene expression was significantly reduced in the liver tissue of female mice consuming a high sucrose solution compared to control groups [50].

Elevated leptin/adiponectin ratios reflect increased inflammation, which contributes to insulin resistance, cardiovascular disease, and T2DM [51]. Our study found that DMSO, which acts as a carrier in an interesting way, increases this ratio. TrkB inhibition, on the other hand, was found to partially reduce this ratio, thereby positively contributing to the inflammatory state (Table 2, Figure 2). It has been demonstrated that BDNF deficiency in mice fed a high‐sucrose diet increases the leptin/adiponectin ratio, thereby causing an inflammatory state [47]. From this perspective, our study suggests that TrkB inhibition may contribute, at least partially, to the inflammatory state Table 3.

**Table 3:** Hepatic BDNF levels of control, sucrose, sucrose+DMSO, and sucrose+ANA‐12 for 4 months.

	Control	Sucrose	Sucrose + DMSO	Sucrose + ANA‐12
*BDNF (ng/mL)*	2.33 (0.51) [2.07–2.58]	1.70 (0.41)^a^ [1.53–1.94]	1.78 (1.14) [1.41–2.56]	1.70 (0.31)^a^ [1.66–1.96]

*Note:* Values are median – (IQR) and quartiles of 25%–75%.

^a^ *p* < 0.05 compared with control.

Ceramide is a lipid derivative that plays a role in the development of insulin resistance by directly inhibiting insulin signaling pathways and lipotoxicity. Ceramide accumulation is often associated with decreased mitochondrial function [52]. Our study demonstrated that DMSO significantly increased serum ceramide levels, while TrkB inhibition decreased serum ceramide concentration (Table 2, Figure 2). A clinical study has shown that higher levels of different types of ceramides contribute to the increased risk of cardiometabolic disease associated with the consumption of sucrose‐containing liquid beverages [53]. A high‐fat and high‐sucrose diet can lead to ceramide accumulation in the liver, and it has been shown that high sucrose, especially when combined with high fat, exacerbates ceramide accumulation through impaired mitochondrial function [54].

**Figure 3:**
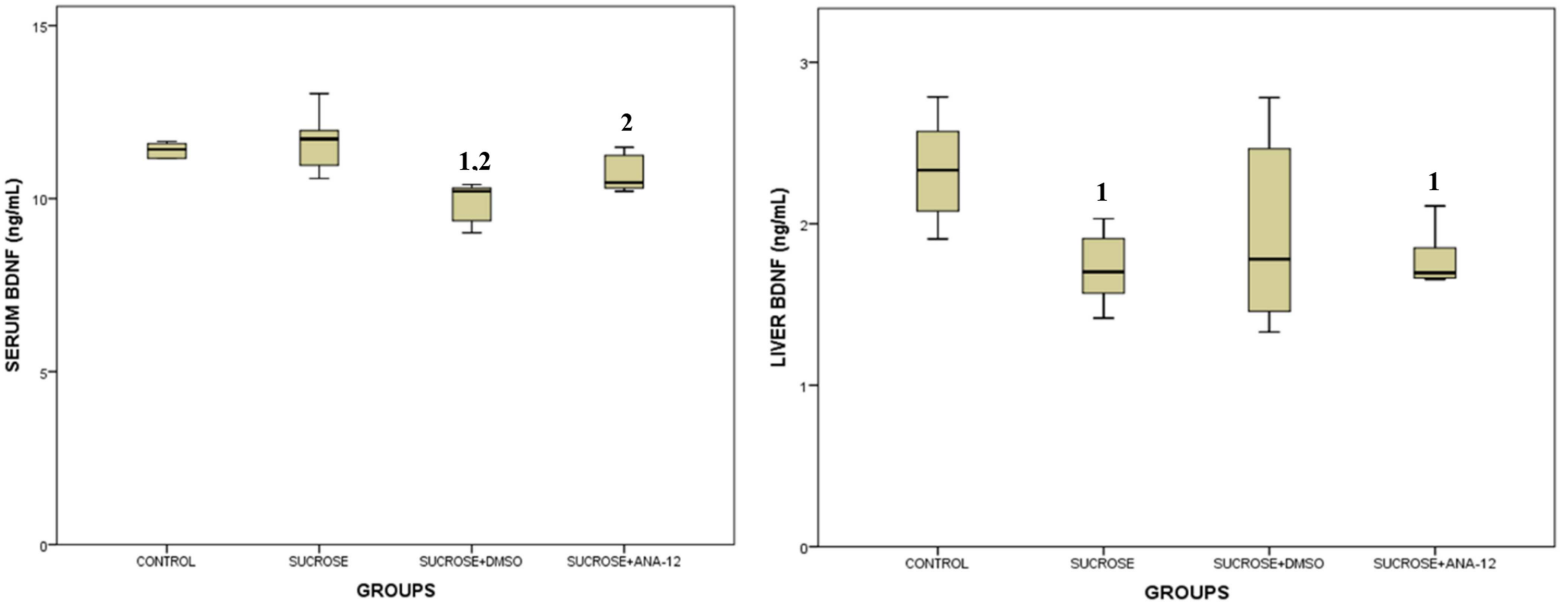
Serum and hepatic BDNF in each group. (^1^ *p* < 0.05 compared with control; ^2^ *p* < 0.05 compared with sucrose).

The brain plays an important role in regulating energy metabolism through the effects of BDNF, lipid oxidation, and mitochondrial functions produced by tissues such as skeletal muscle. Although the functions of BDNF in the liver are less understood, it has the potential to be one of the target molecules for improving hepatic problems [55]. While chronic consumption of high sucrose solution did not significantly alter serum BDNF levels, both DMSO and TrkB inhibition were found to significantly reduce serum BDNF levels. Additionally, high sucrose solution consumption has been found to cause a significant decrease in hepatic BDNF levels (Tables 2 and 3, Figure 3). High consumption of white and brown sugar has been shown to statistically significantly reduce serum BDNF levels in albino rats [56]. While low serum BDNF levels were observed in BDNF heterozygous mice, it was demonstrated that a high sucrose diet did not cause any significant change [13]. The divergent pattern between serum and hepatic BDNF levels should also be interpreted cautiously. In the present study, sucrose exposure was associated with reduced hepatic BDNF levels, whereas serum BDNF was not significantly altered in the sucrose‐only group. This suggests that hepatic BDNF alterations may reflect tissue‐specific responses that are not necessarily mirrored by circulating BDNF concentrations. Moreover, because ANA‐12 was administered systemically, the present design cannot determine whether the observed hepatic mitochondrial enzyme activity and oxidative stress‐related changes resulted from direct hepatic TrkB inhibition or from indirect effects mediated through the central nervous system or other metabolically active peripheral tissues. Therefore, the current findings should be interpreted as associations between TrkB inhibition and hepatic biochemical responses under sucrose‐induced metabolic stress, rather than as evidence of a direct liver‐specific mechanistic pathway.

**Figure 4:**
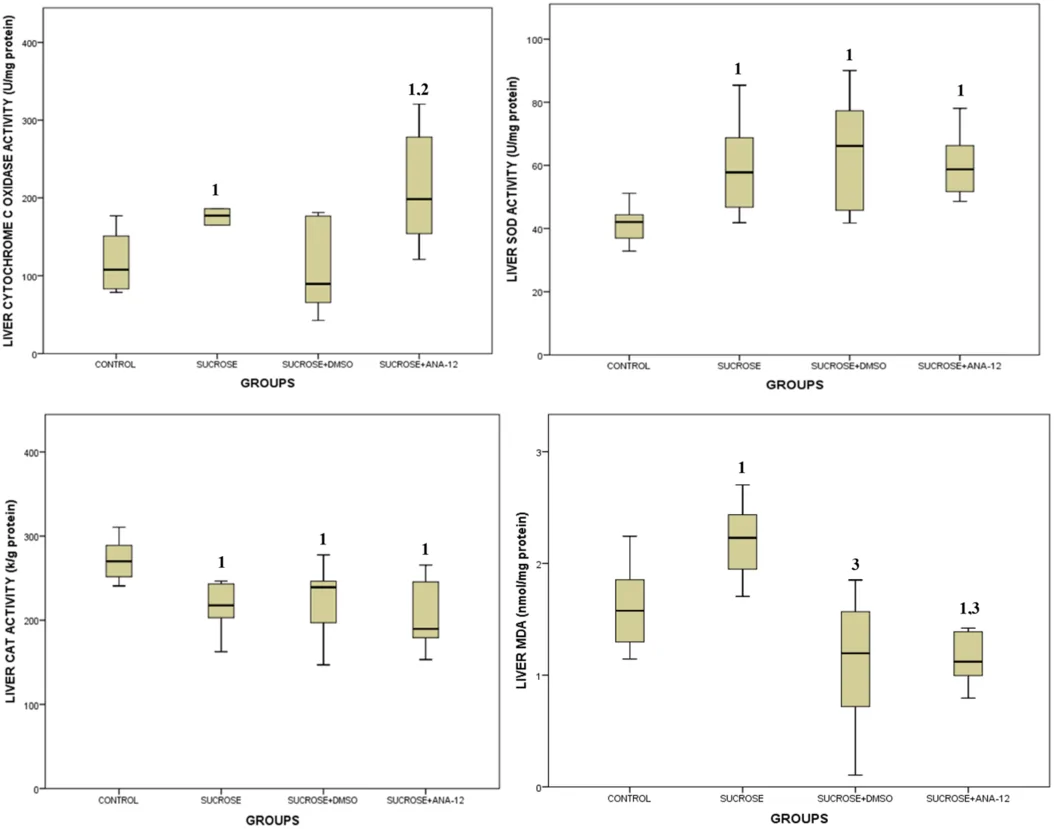
Hepatic cytochrome c oxidase, SOD, and CAT activities and MDA levels in each group. (^1^ *p* < 0.05 compared with control; ^2^ *p* < 0.05 compared with sucrose; ^3^ *p* < 0.05 compared with sucrose+DMSO).

MDA is widely recognized as a reliable biomarker reflecting the extent of lipid peroxidation and oxidative injury [57]. The significant increase in MDA in the sucrose group indicates that a high sucrose diet significantly increases hepatic oxidative stress. It is noteworthy that both DMSO and TrkB inhibition significantly reduced liver MDA levels compared to the sucrose group. Thus, it can be suggested that DMSO or ANA‐12 may be associated with reduced lipid peroxidation‐related biochemical changes under the present experimental conditions (Table 4, Figure 4). SOD functions as a key antioxidant enzyme by catalyzing the dismutation of superoxide anions to hydrogen peroxide [58]. Increased SOD activity in all sucrose groups compared to the control group can be interpreted as a compensatory antioxidant response. It appears that the SOD enzyme may play a role in an active defense mechanism against oxidative stress associated with chronic sucrose consumption (Table 4, Figure 4). In rats fed a 40% sucrose solution for 180 days, an increase in hepatic SOD and MDA levels was observed, which could be an adaptive response to the high oxidative stress associated with sucrose consumption [28]. CAT functions as a key antioxidant enzyme by decomposing hydrogen peroxide into harmless products such as water and molecular oxygen [23]. It has been determined that catalase activity decreases in all groups containing sucrose (Table 4, Figure 4). Hepatic catalase activity was significantly reduced in rats fed a 32% sucrose solution for 16 weeks, highlighting impaired antioxidant defense mechanisms [59]. These results suggest a possible association between BDNF–TrkB‐related signaling and hepatic mitochondrial enzyme activity under insulin‐resistant conditions; however, the current design does not establish a direct causal effect on mitochondrial oxidative capacity or redox homeostasis [7].

**Table 4:** Hepatic cytochrome c oxidase, SOD, and catalase activities, and MDA levels of control, sucrose, sucrose+DMSO, and sucrose+ANA‐12 for 4 months.

	Control	Sucrose	Sucrose + DMSO	Sucrose + ANA‐12
*Cytochrome c oxidase activity* *(U/mg protein)*	107.79 (83.15) [80.88–164.03]	177.24 (63.23)^a^ [150.93–214.16]	89.58 (116.13) [63.27–179.40]	198.33 (183.35)^a^, ^b^ [137.28–320.63]
*SOD activity* *(U/mg protein)*	42.07 (12.42) [32.86–45.28]	57.77 (33.21)^a^ [42.56–75.77]	66.13 (35.04)^a^ [44.81–79.85]	58.72 (16.21)^a^ [50.95–67.15]
*CAT activity* *(k/g protein)*	270.02 (45.36) [248.96–294.32]	217.64 (46.94)^a^ [197.10–244.04]	239.10 (61.42)^a^ [186.97–248.39]	189.75 (77.86)^a^ [172.77–250.63]
*MDA levels* *(nmol/mg protein)*	1.58 (0.71) [1.25–1.96]	2.23 (0.63)^a^ [1.87–2.51]	1.20 (1.01)^c^ [0.56–1.57]	1.12 (0.45)^a^, ^c^ [0.95–1.40]

*Note:* Values are median – (IQR) and quartiles of 25%–75%.

^a^ *p* < 0.05 compared with control.

^b^ *p* < 0.05 compared with sucrose+DMSO.

^c^ *p* < 0.05 compared with sucrose.

Cytochrome c oxidase, known as complex IV, is the final enzyme in the mitochondrial electron transport chain and is critical for cellular respiration [60]. Our study determined that chronic consumption of high sucrose solution and TrkB inhibition increased hepatic cytochrome c oxidase enzyme activity (Table 4, Figure 4). However, because liver histology and direct markers of mitochondrial density were not assessed, these findings should be interpreted as changes in hepatic mitochondrial enzyme activity rather than as definitive evidence of altered mitochondrial abundance or global mitochondrial function. Liver mitochondria were impaired in rats fed a 35% sucrose diet for 20 weeks [61]. Additionally, feeding a diet containing 72% sucrose in solid form resulted in decreased cytochrome c oxidase activity in the liver mitochondria of both diabetic and non‐diabetic rats [62]. In rats fed a 40% sucrose diet for 8 weeks, both a decrease in synthesis and a decrease in liver mitochondrial cytochrome content were determined [63].

Although ANA‐12 administration was associated with reduced hepatic MDA levels and increased cytochrome c oxidase and SOD activities, these findings should not be interpreted as evidence of a uniformly protective metabolic effect. In the present study, ANA‐12 did not significantly improve body weight gain, HOMA‐IR scores, or hypercholesterolemia. Moreover, serum triglyceride levels were significantly increased in the sucrose+ANA‐12 group. These findings indicate that TrkB inhibition may exert parameter‐specific and potentially divergent effects, with partial modulation of hepatic oxidative stress markers but no clear improvement in systemic insulin resistance or lipid homeostasis. Therefore, the effects of ANA‐12 should be interpreted cautiously as biochemical changes observed under chronic sucrose‐induced metabolic stress, rather than as definitive evidence of therapeutic efficacy. In conclusion, this study demonstrates that chronic high sucrose consumption leads to multifaceted metabolic dysfunction accompanied by insulin resistance, serum adipokine imbalance, ceramide accumulation, and oxidative stress. Although the BDNF–TrkB signaling pathway is critically involved in metabolic regulation, its functional role is multifaceted and tightly controlled. TrkB inhibition has been shown to have beneficial effects on some variables, while exhibiting adverse effects on others. Thus, it is suggested that the BDNF‐TrkB signaling pathway requires a multifaceted assessment in metabolic regulation, considering factors such as feeding duration, hormone‐receptor binding capacity, tissue‐specific effects, age, and gender. In future studies, the necessity of separately evaluating the tissue‐specific effects of the BDNF–TrkB pathway (liver, muscle, adipose tissue, and brain) and conducting comparative studies between TrkB agonists and antagonists will contribute to a clearer understanding of the therapeutic potential of the BDNF‐TrkB signaling pathway. It is also important to consider the effects of DMSO, particularly in studies examining glucose metabolism and where it is used as a solvent [36, 64].

## 5 Limitations

The present study has several limitations. First, the absence of ANA‐12‐only and DMSO‐only groups under non‐sucrose‐fed conditions limits the ability to distinguish insulin resistance‐specific effects of TrkB inhibition from basal pharmacological or vehicle‐related effects. This issue is particularly relevant because the Sucrose+DMSO group showed distinct changes in several biochemical parameters. Second, the sucrose exposure period and ANA‐12 administration were not equivalent in duration; sucrose exposure was maintained for 16 weeks, whereas ANA‐12 was administered only during the final 21 days. Therefore, the findings should be interpreted as the effects of subchronic TrkB inhibition after chronic sucrose‐induced metabolic stress, rather than as the effects of continuous or chronic TrkB inhibition. Third, only male mice were used to reduce variability related to estrous cycle‐associated hormonal fluctuations; however, this design prevents evaluation of sex‐specific responses. Accordingly, future studies should include ANA‐12‐only and DMSO‐only control groups, different treatment durations, and both male and female animals to clarify the basal, vehicle‐related, time‐dependent, and sex‐specific effects of TrkB inhibition.

## Author Contributions

**Akın Bodur:** conceptualization, investigation, methodology, writing – review and editing, writing – original draft, software, formal analysis, project administration, data curation, supervision, resources, funding acquisition. **Hüseyin Çınar Zihni:** methodology, writing – original draft, investigation, writing – review and editing. **Katip Korkmaz:** methodology, investigation, writing – original draft, writing – review and editing. **Solmaz Bodur:** writing – original draft, writing – review and editing, investigation, supervision. **Ahmet Alver:** validation, visualization, writing – review and editing, supervision.

## Conflicts of Interest

The authors declare no conflicts of interest.

## Data Availability

The data that support the findings of this study are available from the corresponding author upon reasonable request.
